# Phenomenological models of Na_V_1.5. A side by side, procedural, hands-on comparison between Hodgkin-Huxley and kinetic formalisms

**DOI:** 10.1038/s41598-019-53662-9

**Published:** 2019-11-25

**Authors:** Emilio Andreozzi, Ilaria Carannante, Giovanni D’Addio, Mario Cesarelli, Pietro Balbi

**Affiliations:** 1 Department of Electrical Engineering and Information Technologies (DIETI), University of Naples Federico II, Naples, Italy; 2 Department of Computational Science and Technology, School of Electrical Engineering and Computer Science, KTH The Royal Institute of Technology, Stockholm, Sweden; 3 Istituti Clinici Scientifici Maugeri IRCCS, Department of Bioengineering of Telese Terme Institute, Telese Terme (BN), Italy; 4 Istituti Clinici Scientifici Maugeri IRCCS, Laboratory of Computational Neurophysiology of Telese Terme Institute, Telese Terme (BN), Italy

**Keywords:** Biophysical models, Computational neuroscience

## Abstract

Computational models of ion channels represent the building blocks of conductance-based, biologically inspired models of neurons and neural networks. Ion channels are still widely modelled by means of the formalism developed by the seminal work of Hodgkin and Huxley (HH), although the electrophysiological features of the channels are currently known to be better fitted by means of kinetic Markov-type models. The present study is aimed at showing why simplified Markov-type kinetic models are more suitable for ion channels modelling as compared to HH ones, and how a manual optimization process can be rationally carried out for both. Previously published experimental data of an illustrative ion channel (Na_V_1.5) are exploited to develop a step by step optimization of the two models in close comparison. A conflicting practical limitation is recognized for the HH model, which only supplies one parameter to model two distinct electrophysiological behaviours. In addition, a step by step procedure is provided to correctly optimize the kinetic Markov-type model. Simplified Markov-type kinetic models are currently the best option to closely approximate the known complexity of the macroscopic currents of ion channels. Their optimization can be achieved through a rationally guided procedure, and allows to obtain models with a computational burden that is comparable with HH models one.

## Introduction

Nowadays, biologically inspired neural simulations, sustained by both escalating computational power and more detailed comprehension of the nervous system physiology^1^, are becoming increasingly popular and appreciated, despite their concurrent boosting complexity^2–7^.

At the core of those simulations, multi-compartmental, conductance-based models of single neural cells can be retrieved with variable degrees of morphological and biophysical details. The modelled single cells in turn mainly and directly derive their electrophysiological properties (that is, the fundamentals of the entire modelled neural networks) from the kinetics of the macroscopic currents of different kinds of voltage-gated ion channels^8^.

Phenomenological models of ion channels, therefore, constitute the building blocks of biologically inspired neuronal cells and neural networks models.

Current electrophysiological techniques are able to provide huge amount of data with unprecedented details on voltage-gated ion channels. These techniques have been developed as patch-clamp methods^9^ but, when used in whole-cell configuration, result more suitable for recording the macroscopic currents of ion channels, significantly increasing our comprehension of the kinetic features of ion channels.

Yet, a gap in integrating the improved access to functional properties of ion channels into whole-cell models has been for long recognized^10,11^.

Until recently the phenomenological behaviour of the voltage-gated ion channels has been mainly modelled according to the seminal work of Hodgkin and Huxley^12^. The availability of analytical solutions for Hodgkin and Huxley (HH) equations, which can be directly fitted to the experimental results, makes the HH formalism a gold standard for ion channels modelling. In addition, the reasonable computational burden of HH models makes them particularly suitable for the implementation in biologically inspired neural networks of increasing complexity. However, the HH formalism turned out to carry theoretical and practical limitations in reproducing the complex electrophysiological behaviour of ion channels^11,13–16^.

On the other hand, Markov-type kinetic models, characterized by a set of not independent states with transitions between states governed by barrier-style equations, proved capable to approximate with higher accuracy the known complexity of the voltage-gated ion channels^17–20^. Markov-type kinetic models can also be designed to reproduce the behaviour of a single channel protein, with each state corresponding to a specific physical conformation of the protein. These extremely detailed models, however, need a huge number of states (e.g., ^21^), resulting in a heavy computational load, which makes them unsuitable for implementation in neural networks.

Thus, simplified Markov-type kinetic models have been proposed^17,19^ to provide efficient modelling of ion-channels behaviour, by overcoming the limitations of the HH formalism, while keeping the computational burden sufficiently low to make them suitable for implementation in biologically inspired neural networks.

They are built with a reduced number of states and addressed to deterministically reproduce the macroscopic current kinetics of ion channels, rather than their conformational changes.

In this work, a point-by-point comparison between HH and Markov-type kinetic models of an illustrative human sodium channel (Na_V_1.5) is performed, with the aim of clarifying the process of optimization of a simplified kinetic model in close relationship with a correspondent HH model. The study unveils the limits of HH models and suggests simplified Markov-type kinetic models as an essential tool to *in silico* approximate the known complexity of the ion channels kinetics.

A critical limitation of HH formalism is recognized from the simultaneous optimization of the steady-state availability and the recovery from fast inactivation, which led to a flawed modelling of the electrophysiological features of the channel.

## Methods

### Experimental data of Na_V_1.5^22^

We chose to simulate the electrophysiological behaviour of Na_V_1.5 because quite comprehensive experimental data for this channel have been already published and made available to be shared and reused under a Creative Common license^22^.

Experimental data on Na_V_1.5 macroscopic currents were obtained by heterologously expressing the α-subunit of the ion channel in a mammalian cell line (Human Embryonic Kidney 293 cells). No β-subunits were co-expressed in the study and the electrophysiological experiments were conducted by means of the whole-cell patch-clamp method at room temperature^22^.

Na_V_1.5 is the isoform of the sodium channel α-subunit typically expressed in the heart, where it is mainly involved in the cardiac rhythmogenesis, as revealed by the rare channel mutations responsible for severe genetic arrythmiae^22,23^. But Na_V_1.5 were also detected in different structures of the brain^24^, where they clustered at a high density in the neuronal processes, mainly axons.

### The HH model

According to the original formulation of Hodgkin and Huxley^12,17^, the HH model describes the electrical behavior of an isopotential compartment of the cell membrane, detailing the contributions of the three ionic currents and the membrane capacitance, based on the following differential equation:

1a $${C}_{m}\frac{dV}{dt}=-{g}_{L}(V-{E}_{L})-{g}_{Na}(V)(V-{E}_{Na})-{g}_{K}(V)(V-{E}_{K})$$

where *C*_*m*_ is the membrane capacitance, *V* is the membrane potential, *g*_*Na*_, *g*_*K*_ and *g*_*L*_ are the membrane conductances for *Na*^+^, *K*^+^ and leak currents, respectively, *E*_*Na*_, *E*_*K*_ and *E*_*L*_ are their respective reversal potentials.

The hypothesis of Hodgkin and Huxley was that ionic currents result from the synergic actions of several independent gating particles, which must remain bound to a specific site in the membrane in order to allow ions flow^12^. Each gating particle carries a net electronic charge which allows the membrane potential to switch its position from the inside to the outside or viceversa.

Therefore, the inside-outside transitions are voltage-dependent, as depicted in the following diagram:

$$(outside)\,\underset{\mathop{\longleftarrow }\limits_{{\beta }_{m}(V)}}{\overset{{\alpha }_{m}(V)}{\longrightarrow }}(inside)$$

where the transitions between the outside and inside positions in the membrane depend on the forward and backward rate constants *α* and *β*. By defining *m* as the fraction of particle in the inside position, and (*1-m*) as the fraction outside the membrane, the first-order kinetic equation is obtained:

1b $$\frac{dm}{dt}={\alpha }_{m}(V)\,(1-m)-{\beta }_{m}(V)m$$

Assuming that particles must occupy the inside position to conduct ions, then the conductance must be proportional to some function of *m*. Hodgkin and Huxley^12^ found that the best fit for the nonlinear behavior of the *Na*^+^ current measured in the squid giant axon, could be achieved by assuming for the conductance the following expression:

1c $${g}_{Na}={\bar{g}}_{Na}{m}^{3}h$$

where *m* and *h* are the fractions of two different types of gating particles, and ${\bar{g}}_{Na}$ is the maximum conductance. The interpretation given to this relationship was that, in order to flow through the cell membrane, the *Na*^+^ ions require the gathering of three *m* gating particles and one *h* gating particle, which act independently of each other, thus leading to the *m*^3^*h* form.

Many years later, further studies led to the understanding that ionic currents are caused by the opening and closing of ion channels, with gates within their pores playing the same role of the gating particles. Therefore, the expression of ions conductance provided by Hodgkin and Huxley was reinterpreted by assuming that the ions flow through the channel’s pore is regulated by the independent openings and closings of four internal voltage-sensitive gates, in such a way that ions conduction is possible only when all gates are open.

The rate constants of *m* have a functional dependence on membrane potential that promotes gate opening in response to depolarization, in order to reproduce a phenomenon referred to as *activation*. On the other hand, the rate constants of *h* have a functional dependence on membrane potential that, in response to depolarization, promotes gate closing (i.e. entire channel closing, as just one closed gate prevents ions flow), thus allowing to model the dynamics of another phenomenon referred to as *inactivation*.

Thus, the set of differential equations (HH equations) which model the main features of the *Na*^+^ current is composed by the (1b) and the following one:

1d $$\frac{dh}{dt}={\alpha }_{h}(V)(1-h)-{\beta }_{h}(V)h$$

The rate constants of *m* and *h* were estimated by fitting empirical functions of membrane potential to the experimental data:

1e $${\alpha }_{m}=\frac{0.1\cdot (V+25)}{{e}^{\frac{(V+25)}{10}}-\,1}$$

1f $${\beta }_{m}=4\cdot {e}^{\frac{(V)}{18}}$$

1g $${\alpha }_{h}=0.07\cdot {e}^{\frac{(V)}{20}}$$

1h $${\beta }_{h}=\frac{1}{1+{e}^{\frac{(V+30)}{10}}}$$

These functions were estimated at a temperature of 6 °C and the voltage axis was reversed in polarity (voltage values were given with respect to the resting membrane potential).

**Figure 1:**
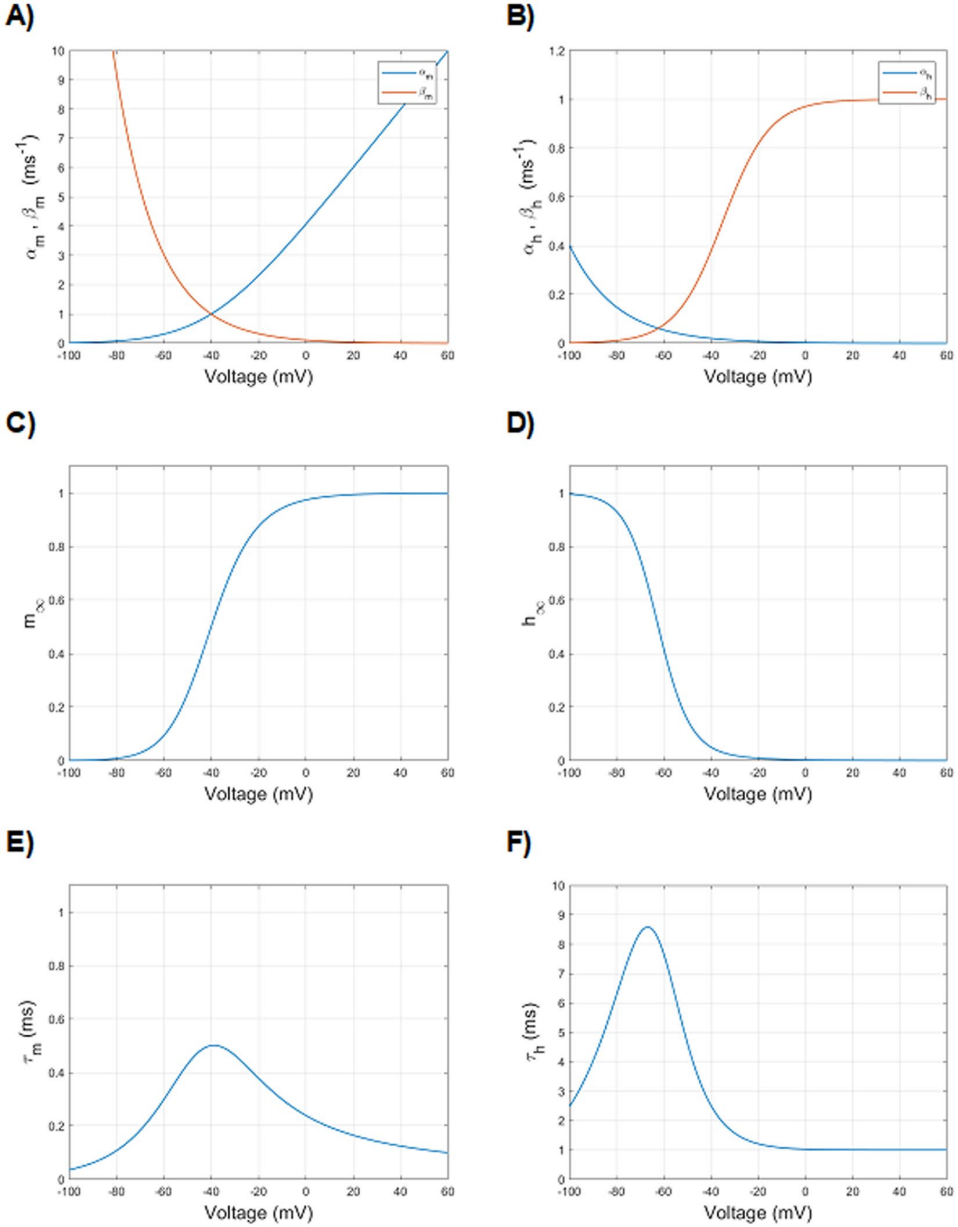
Voltage dependence of particle (gate) variables and rate constants of sodium ion channel of *Loligo*, as found in the original paper by Hodgkin and Huxley^12^, redrawn according to the actual notation on voltage axis direction and rest potential reference. (**A**) Voltage dependence of forward (α_*m*_, blue) and backward (${\beta }_{m},\,\,$red) rate constants of activation. (**B**) Voltage dependence of forward (α_*h*_, blue) and backward (${\beta }_{h}$, red) rate constants of inactivation. (**C**) Voltage dependence of steady-state activation (${m}_{\infty }$). (**D**) Voltage dependence of steady-state inactivation (${h}_{\infty }$). (**E**) Voltage dependence of activation time constant (${\tau }_{m}$). (**F**) Voltage dependence of inactivation time constant (${\tau }_{h}$).

By adopting the modern convention on voltage axis orientation and a resting voltage of −65 mV, the HH equations can be reworked as follows:

1i $${\alpha }_{m}=A\cdot \frac{-(V-({V}_{1/2}))}{{e}^{\frac{(V-({V}_{1/2}))}{k}}-1}$$

1j $${\beta }_{m}=A\cdot {e}^{\frac{(V-({V}_{1/2}))}{k}}$$

1k $${\alpha }_{h}=A\cdot {e}^{\frac{(V-({V}_{1/2}))}{k}}$$

1l $${\beta }_{h}=\frac{A}{1+{e}^{\frac{(V-({V}_{1/2}))}{k}}}$$

with *A*, *V*_1/2_, *k* being the parameters that determine the voltage-dependence of the rate constants. The values for these parameters, reported in Table 1, allow to replicate the original Hodgkin and Huxley^12^ experimental results (Fig. 1A,B).

**Table 1:** Parameters of the Hodkin and Huxley rate constants equations (Eq. 1i–1l), rewritten according to the current conventions.

	*α*_m_	*β*_*m*_	*α*_*h*_	*β*_*h*_
A	0.1	4	0.07	1
V_1/2_	−40	−65	−65	−35
k	10	−18	−20	−10

The HH model is often written in the following equivalent form, which generally results more convenient to fit the experimental data:

1m $$\frac{dm}{dt}=\frac{1}{{\tau }_{m}(V)}({m}_{\infty }(V)-m)$$

1n $$\frac{dh}{dt}=\frac{1}{{\tau }_{h}(V)}({h}_{\infty }(V)-h)$$

where

1o $${m}_{\infty }=\frac{\alpha m(V)}{\alpha m(V)+\beta m(V)}$$

1p $${\tau }_{m}=\frac{1}{\alpha m(V)+\beta m(V)}$$

1q $${h}_{\infty }=\frac{\alpha h(V)}{\alpha h(V)+\beta h(V)}$$

1r $${\tau }_{h}=\frac{1}{\alpha h(V)+\beta h(V)}$$

Here, ${m}_{\infty }$ is the value approached by *m* at the steady-state, referred to as steady-state activation, and ${\tau }_{m}$ is the activation time constant of *Na*^+^ current (Fig. 1C,E). Analogously, is it possible to define for *h* the steady-state inactivation ${h}_{\infty }$ and the inactivation time constant ${\tau }_{h}$ (Fig. 1D,F).

### The Markov-type kinetic model

The methods of kinetic model mathematical implementation, the considered electrophysiological protocols and their *in silico* simulation, the fitting procedure, the implementation of the channel models in a neuron model, have all been previously described^25^, and will be here reported in detail.

**Figure 2:**
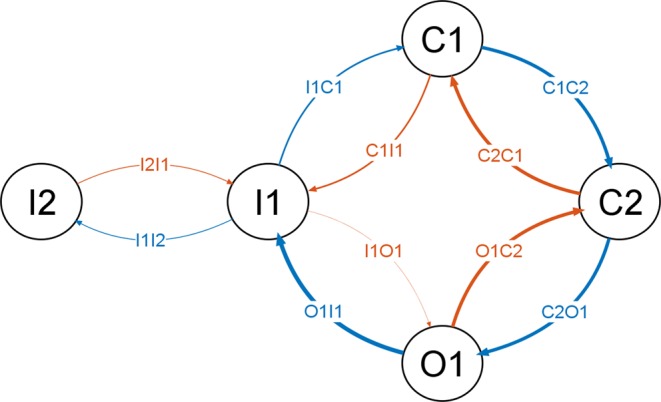
Diagram of the five-state kinetic Markov-type model. The thickness of the transitions is drawn as a schematic cue to the maximal amplitude of the transition rate.

The Markov-type kinetic model is characterized by a five-state diagram, with one open, two closed and two inactivated states (Fig. 2).

The second inactivated state (I2) is considered as a deeper inactivated state, and it is connected only to the first inactivated state (I1).

Apart from the transitions between O1 and I1, all other transitions between two consecutive states are considered reversible, because the numerical values of the paired forward and backward transitions are of the same order of magnitude. On the other hand, the transition O1 to I1 can be considered irreversible, given the extremely small rate value of the backward transition (I1 to O1).

The dynamics of the fractions of channels being in the five different states (later referred to as the “states”, for the sake of simplicity) are described by five coupled ordinary differential equations, as follows:

2a $$\frac{dC1}{dt}=I1C1\,\ast \,I1+C2C1\,\ast \,C2-(C1C2+C1I1)\,\ast \,C1$$

2b $$\frac{dC2}{dt}=C1C2\,\ast \,C1+O1C2\,\ast \,O1-(C2C1+C2O1)\,\ast \,C2$$

2c $$\frac{dO1}{dt}=C2O1\,\ast \,C2+I1O1\,\ast \,I1-(O1C2+O1I1)\,\ast \,O1$$

2d $$\frac{dI1}{dt}=I2I1\,\ast \,I2+C1I1\,\ast \,C1+O1I1\,\ast \,O1-(I1C1+I1I2+I1O1)\,\ast \,I1$$

2e $$\frac{dI2}{dt}=I1I2\,\ast \,I1-I2I1\,\ast \,I2$$

Moreover, the states obey the law of mass conservation:

2f $$O1+I1+I2+C1+C2=1$$

Ohm’s law governs the ion channel curents, and the fraction of channels in the open state determines the channel conductance, with the sodium maximal conductance being the proportionality coefficient, according to the following equation:

2g $${I}_{Na}(t)={\bar{g}}_{Na}\cdot O1(t)\cdot (V(t)-{E}_{Na})$$

where ${\bar{g}}_{Na}$ is the sodium maximal conductance, O1 is the fraction of channels in the open state (bounded between 0 and 1) and *E*_*Na*_ is the reversal potential of the sodium ion.

Previous studies^12,34^ assumed that the rate constants dependence on membrane voltage can be modelled as an exponential function, a sigmoidal function, or a combined linear and exponential function (Fig. 1A,B).

In other cases, according to the theoretical approach of the thermodynamic theory^17,18^, a sigmoidal curve with minimum and maximum asymptotes has been adopted, which was described by the following equation

2h $${A}_{\omega }={\tau }_{min}^{\omega }+{\tau }_{max}^{\omega }\cdot {[1+exp(\frac{V-{V}_{1/2}^{\omega }}{{k}^{\omega }})]}^{-1}$$

where *ω* is the transition between two states, ${\tau }_{min}^{\omega }$ and ${\tau }_{max}^{\omega }\,$are the two asymptotes, ${V}_{1/2}^{\omega }$ the hemiactivation voltage, and ${k}^{\omega }$ the slope which describes the voltage sensitivity of the transition rate.

**Figure 3:**
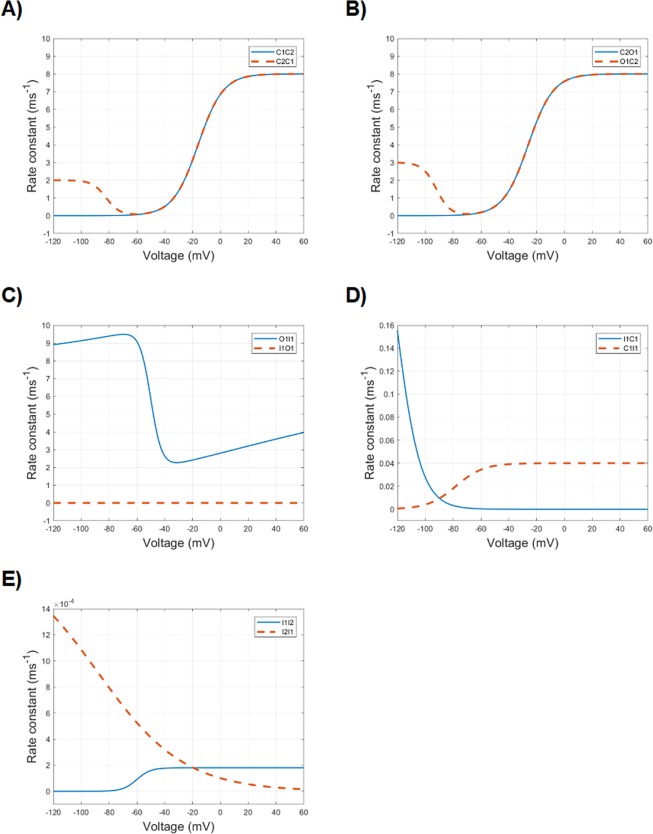
Voltage dependence of the following ten transition rates of the kinetic Markov-type model: (**A**) C1C2 (solid blue) and C2C1 (dashed red); (**B**) C2O1 (solid blue) and O1C2 (dashed red); (**C**) O1I1 (solid blue) and I1O1 (dashed red); (**D**) I1C1 (solid blue) and C1I1 (dashed red); (**E**) I1I2 (solid blue) and I2I1 (dashed red). Note the different ordinate scales.

In a previous work^25^ we found that a sigmoidal function is particularly well suited for setting up with accuracy the model parameters. The sigmoid has the minimum asymptote set to zero for most of the transitions, but for the O1 to I1 transition, where a non-zero value is adopted. In addition, a bending before the rising slope of the curve is added, in order to accurately account for the time course of the current-voltage curves (Fig. 3A–I).

Thus, the modified sigmoid is mathematically represented as a combination of two opposite sigmoids: the first one carrying a positive slope factor and adopted to describe the transition rate for more polarized voltages, the second one characterized by a negative slope factor and used for more depolarized voltages.

As a result, the general equation adopted to describe this double sigmoid was set as follows:

2i $${A}_{\omega }={B}_{hyp}^{\omega }\cdot {[1+{e}^{(\frac{V-{V}_{hyp}^{\omega }}{{k}_{hyp}^{\omega }})}]}^{-1}+{B}_{dep}^{\omega }\cdot {[1+{e}^{(\frac{V-{V}_{dep}^{\omega }}{{k}_{dep}^{\omega }})}]}^{-1}$$

where ${B}_{hyp}^{\omega }$, ${V}_{hyp}^{\omega }$ and ${k}_{hyp}^{\omega }$ are, respectively, the magnitude, the hemiactivation and the slope factor, of the voltage dependence of the transition rate *ω* in the hyperpolarized region, and ${B}_{dep}^{\omega }$, ${V}_{dep}^{\omega }$ and ${k}_{dep}^{\omega }$ are the corresponding values in the depolarized region.

According to this formalism, a positive value is assigned to the slope factor (*k*) in the hyperpolarized range and a negative value in the depolarized one. In addition, one of the two terms of the Eq. (2i) can be dropped when the transition rate is described by a simple sigmoid.

### The electrophysiological protocols

#### Activation curves and normalized conductance-voltage dependence

**Figure 4:**
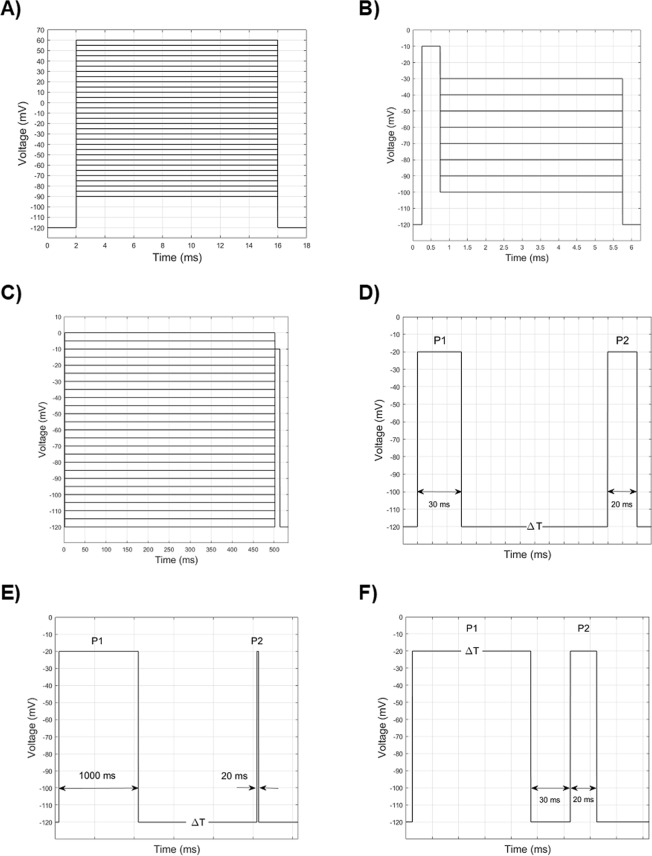
Experimental and simulated voltage clamp protocols. (**A**) Activation: A 2-ms pulse at −120 mV is followed by a series of 14 ms long depolarizations (from −90 to +60 mV), in steps of 5 mV. (**B**) Deactivation: After a 0.5-ms pulse at −120 mV, a 0.5-ms depolarization at −10 mV is delivered, followed by a series of 5-ms long repolarization, from −100 to −30 mV, in steps of 10 mV. (**C**) Steady-state availability: Following a series of 500-ms long conditioning depolarizations from −120 to 0 mV in steps of 5 mV, a 20-ms long test stimulus at −10 mV is delivered. (**D**) Recovery from fast inactivation: After a 30-ms long conditioning depolarization at −20 mV (P1), variable time intervals (from 0.1 to 1000 ms) of repolarization at −120 mV are followed by a 20-ms long probing depolarization at −20 mV (P2). (**E**) Recovery from slow inactivation: similar to the previous protocol, with the difference that the conditioning pulse (P1) is 1000 ms long, and the repolarization time intervals are from 0.1 ms to 7000 ms. (**F**) Development (onset) of slow inactivation: A series of depolarizations at −20 mV of increasing duration, from 10 to 10000 ms (P1), is followed by a brief (30 ms) repolarization impulse at −120 mV and then by a 20-ms long test depolarization at −20 mV (P2).

Voltage-clamp intensity-voltage (activation) curves are obtained by sequentially clamping the channel membrane potential in steps of 5 or 10 mV from a resting value (Fig. 4A).

Normalized conductance-voltage relationship is obtained by converting the current peak values into the respective conductance values, according to the Eq. (3a),

3a $$G=I/(V-{E}_{Na})$$

where *G* is the conductance, *I* is the peak current, *V* is the membrane voltage, *E*_*Na*_ is the sodium equilibrium potential, and *V-E*_*Na*_ is referred to as the driving force. The so evaluated conductance is plotted against voltage clamp values, and the conductance-voltage curves are usually fitted to a Boltzmann Eq. (3b):

3b $$G/{G}_{max}={(1+{e}^{\frac{V-{V}_{\frac{1}{2}}}{k}})}^{-1}$$

where *G*_*max*_ is the maximum conductance, *V*_1*/*2_ is the hemi-activation voltage and *k* is the slope factor.

For each single activation curve, the time constants of activation and inactivation are calculated. The former is evaluated from the onset of the current to its peak, while the latter from the peak to the steady-state (decay from activation). According to the work by Hodgkin and Huxley^12^, the time constants of activation and inactivation are derived by fitting the entire simulated curve to the following equation:

3c $$y=A\,\ast \,{(1-{e}^{\frac{-t}{{\tau }_{m}}})}^{3}\,\ast \,{e}^{\frac{-t}{{\tau }_{h}}}$$

where *τ*_*m*_ and *τ*_*h*_ are the time constants of activation and inactivation, respectively. In this case, the activation segment of the curve is fitted by a third power exponential, while the inactivation one (decay) is fitted by a simple exponential.

### Deactivation curves

The so-called tail currents are evoked by a quick repolarization after a brief depolarizing pulse (Fig. 4B). The preliminary brief depolarizing pulse causes a fraction of channels to open, and the following variable repolarization is administered before inactivation is fully deployed. Thus, the protocol is designed to sample, in terms of HH formalism, the return of the fraction of *m* gating particles to low values during repolarization, or, in terms of Markov-type kinetic model, the transition between the open and closed states, a process called deactivation. It is worth noting that deactivation differs from inactivation, as the latter defines the transition between open and inactivated states.

By varying the voltage of the repolarizing pulse, a series of curves of tail currents are obtained which can be fitted to the mono-exponential Eq. (3d)

3d $$y=A\,\ast \,{e}^{\frac{-t}{\tau }}$$

### Voltage dependence of (normalized) current during fast inactivation (steady-state availability)

In steady-state availability protocols (Fig. 4C), conditioning pulses of variable voltage and long duration are administered to reach a steady-state condition, before applying a depolarizing test stimulus of fixed amplitude. The conditioning stimulus sets a variable fraction of channels into a steady inactivated state depending on its voltage value, while the following depolarizing test stimulus samples the fraction of channels which are still available to open. It is worth mentioning that the inactivation occurs even before the activation threshold is reached. In other words, depolarizing stimuli below the activation threshold are able to move a fraction of channels from closed to inactivated states, without passing through the open state. The normalized current peaks following the test stimuli are plotted against the voltage of the conditioning stimuli, and the resulting curve is fitted to the Boltzmann Eq. (3e)

3e $$I/{I}_{max}=A+(1-A)\cdot {(1+{e}^{\frac{V-{V}_{\frac{1}{2}}}{k}})}^{-1}$$

where *I* is the peak current, *I*_*max*_ is the maximal peak current, *A* is the fraction of non-inactivating channels, *V*_1*/*2_ is the voltage at which half of the channels are inactivated, and *k* is the slope factor.

### Recovery from fast inactivation (repriming)

The recovery from inactivation (or repriming) is sampled by a first (conditioning) depolarizing pulse (P1) of fixed voltage, followed by a variable time interval of repolarization (from tenths to hundreds of milliseconds, in the case of Na_V_1.5^22^), and then by a second (test) depolarizing pulse (P2) of fixed voltage, which samples the fraction of channels that recovered from the inactivation (Fig. 4D) and are available to open. A normalized intensity-time curve is obtained by plotting the current peaks following the test stimuli compared to those following the pre-conditioning stimulus (indicated as *I/Imax* or P2/P1) against the duration of the repolarization interval. The derived curve can be fitted to a single exponential function:

3f $$I/{I}_{max}={A}_{1}(1-{e}^{\frac{-t}{{\tau }_{1}}})$$

### Development of slow inactivation

An initial depolarizing conditioning pulse (P1, −20 mV) of increasing duration (Δt, from 10 ms to 10 s) is followed by a brief (30 ms) repolarizing pulse and then by a second depolarizing test impulse (P2), to probe the fraction of non-inactivated, available channels (Fig. 4E). For short durations, the conditioning pulse initially is able to put a fraction of the channels into the (fast) inactivation. This inactivation features fast kinetics and, in fact, the short duration of the repolarizing pulse is long enough for the channels to recover into non-inactivated states; the second test pulse, then, evokes a full amplitude current. However, for increasing durations of the conditioning pulse P1, the test pulse P2 evokes progressively smaller currents. This phenomenon is commonly interpreted by admitting that progressively longer conditioning tests are able to move a fraction of the channels into a deeper inactivated state with slower kinetics (both for entry and recovery), thus making these channels not available to be opened by the probing test pulse.

The obtained curve is fitted to a single exponential equation (14):

3g $${I}_{2}/{I}_{1}={A}_{1}+{A}_{2}\,\ast \,{e}^{\frac{-t}{\tau }}$$

### Recovery from slow inactivation

Similarly to the fast inactivation, also the recovery from slow inactivation is sampled by means of a double pulse protocol (Fig. 4D), in which the first depolarizing conditioning pulse (P1) is followed by a repolarizing time interval of increasing duration (Δt, from 0.1 s to 10 s), followed by a short probing depolarization (P2). The difference with the fast inactivation protocol is that P1 is much longer (1000 ms, compared to 100 ms).

The obtained curve is fitted to a double exponential function (15):

3h $$I/{I}_{max}={A}_{1}(1-{e}^{\frac{-t}{{\tau }_{1}}})+{A}_{2}(1-{e}^{\frac{-t}{{\tau }_{2}}})$$

where *A*_*1*_ and *A*_*2*_ are proportional coefficients, *t* is the time, *τ*_*1*_ and *τ*_*2*_ are the fast and slow recovery time constants, respectively.

### Simulations of the experimental procedure in NEURON

The in silico experiments were developed and performed with the simulation environment NEURON version 7.6^26^. The NMODL language of NEURON, a derivative of the SCoP package^27^, was exploited to write and solve the differential equations of the channel models.

It is worth observing that in NMODL different setups and methods can be used to solve the models. For example, in the DERIVATIVE block the differential equations of the model are explicitly specified, as opposed to the KINETIC block where the model is described in terms of chemical reactions equations. This is very convenient when the model contains several reactions, although it slows down the simulations. In the present study we preferred to use the same block (DERIVATIVE block) for both models, and the same numerical method to solve them, in order to achieve a reliable comparison.

All experiments were performed on a one-compartmental 50 μm long cylindrical ‘soma’ with a diameter of 63.66 μm, so that the membrane area was set to 10’000 μm^2^. The membrane capacitance^28^ was set to 1 μF/cm^2^. The maximal conductance density for each voltage-gated sodium channel isomer inserted into the soma was arbitrarily set to 0.1 S/cm^2^, and the resulting ionic current density was measured in mA/cm^2^. The capacitive currents were subtracted from the total current in all the simulations. The single integration time step (dt) was set to 0.025 ms for all simulations, except for the running time tests, where it was set to 0.001 ms. The virtual electrode for the voltage clamp experiments was located into the soma.

The thermal sensitivity of any biological process can be described by its temperature coefficient (Q10). Classically, it is defined as the ratio of a reaction rate measured at two temperatures 10 degrees apart^29^. In ion channel research, current amplitudes or time constants are often used to calculate Q10 value, instead of reaction rates. A single value of Q10 is typically reported to indicate the temperature dependence of a channel. In the present study, at every step the rate constants of each transition were multiplied by the temperature coefficient, Q10, calculated as follows:

3i $${Q}_{10}={3}^{(\frac{{T}^{^\circ }-{20}^{^\circ }}{{10}^{^\circ }})}$$

Original NEURON source code was developed to simulate the protocols needed to yield the electrophysiological features of the channels.

The source code along with the virtual experimental procedures is available as a ModelDB^30^ entry (Access Number: 257747).

The simulations were performed on an iMac desktop computer running a MacOS version 10.14.3 (™ and © 1983 – 2017, Apple Inc, Cupertino, CA, USA) and on a Tuxedo laptop with processor Intel® Core™ i7-7700HQ and 16GB of RAM running Ubuntu 18.04.2 LTS.

### Fitting the experimental data with the models

For modellers, the process of fitting the experimental data may significantly differ according to the availability of raw electrophysiological data (the single electrophysiological curves of different protocols).

The procedure devised by Hodgkin and Huxley^12^ can be applied to HH models, when raw data are available. In this case, the ${m}_{\infty }$, ${\tau }_{m}$, ${h}_{\infty }$ and ${\tau }_{h}$ variables can be directly derived from the single curves of activation, with some assumptions, and the rate constants α_*m*_, β_*m*_, α_*h*_ and β_*h*_ can be calculated by rearranging the Eq. (1o–1r). Afterwards, each single rate constant can be plotted against the voltage and fitted to the original HH expressions (1i) to (1l). Finally, a direct superimposition of real and simulated traces provides the best evidence of the good fit of the model to the experimental data.

However, modellers are rarely provided with raw electrophysiological data, so in most cases it is not possible to directly derive the ${m}_{\infty }$, ${\tau }_{m}$, ${h}_{\infty }$ and ${\tau }_{h}$ variables. In these cases, channels modelling relies on indirect data about the kinetics of the channels, such as the normalized conductance-voltage relationship, the steady-state availability curve, the repriming curve, etc. The research of the best fit is carried out by empirically tuning the parameters of the expressions (1i) to (1l), in order to estimate the parameters of the Eqs. (3b–h), which carry substantial information on the kinetics of the macroscopic currents of the channel.

The same procedure is also usually performed for Markov-type kinetic models and has been performed in the present study as well.

### Fitting procedure in NEURON

Each simulated electrophysiological protocol in turn had the appropriate plot supplied by the developed code. The plots provided the macroscopic currents and the electrophysiological relationships to be compared with the experimental ones. The experimental curves and relationships were firstly compared with the simulated ones by means of visual ispection. Then, the modelled curves were fitted to the Eqs. (3b–h), as appropriate, by means of a nonlinear least-squares minimization method available in NEURON (Multiple Run Fitter subroutine), derived from the PRAXIS method^31^. Finally, the parameters of the Eqs. (3b–h) of the modelled curves were compared to those of the experimental curves (Table 1). The agreement between modelled and experimental data was considered acceptable when the former resulted to be within two standard deviations of the latter.

### Implementation of the channel models in a neuron model

The suitability for the implementation in whole-cell models of both HH and Markov-type kinetic models was assessed by comparing the features of the spikes they were able to provide, along with their running times and computational loads. To this aim, we inserted the developed channel models in a previously published cell model^32^, on which we performed a series of simulations involving virtual voltage-clamp and current stimulation experiments.

The previously published cell model was downloaded from the ModelDB^30^ repository (accessed on February 15^th^ 2019), where it is accessible with the accession number: 3805.

## Results

Direct comparison of all electrophysiological features of both HH and kinetic Markov-type models with the experimental data^22^ is provided in Table 2. The displayed parameters supply the best fit to the real data for both models.

The sequential procedures adopted to achieve the best fitting parameters are described in the following paragraphs.

### Activation

#### HH model

At the resting polarized potential, the rate constants of inactivation α_*h*_ and *β*_*h*_ (Fig. 1B) make the value of *h* close to 1 (that is, no inactivation: Fig. 1D). However, the channel is not conducting, as the rate constants of activation make the value of *m* equal to 0 (Fig. 1A,C). By stepping the potential to more depolarized values, *m* grows rapidly and the channel starts conducting ions. At the same time, due to the predominance of backward rate constant of inactivation at depolarized values (Fig. 1B), *h* begins to decrease till to a value equal to 0 (inactivation sets in, Fig. 1D). However, due to the slower development of inactivation compared to the activation (smaller values of rate constants of *h* compared to those of *m*), inactivation sets in with a delay, which is responsible for the sodium current peak during activation curves.

**Figure 5:**
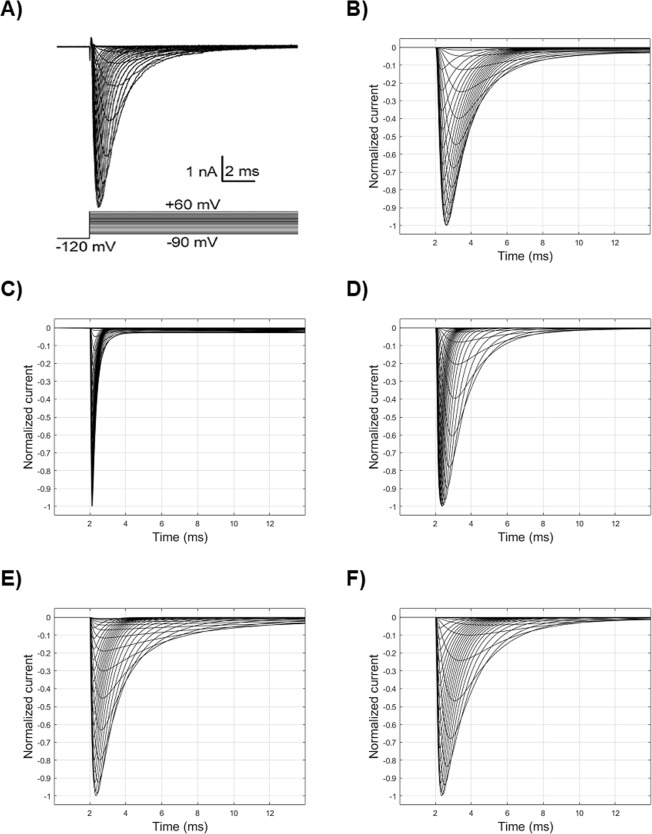
Real and simulated sodium current curves following the activation voltage clamp. (**A**) Real curves from a previously published experimental study^22^, recorded at 25 °C. (**B**) Simulated curves obtained by the original HH model^12^, at 6 °C (Table 2a). (**C**) Simulated curves obtained by the original HH model, at 25 °C. (**D**) Simulated curves after modifying the original HH model (Table 3b). (**E**) Simulated curves obtained by a kinetic Markov-type model with 4 states: one closed, one open and two inactivated states. (**F**) Simulated curves obtained by a kinetic Markov-type model with 5 states.

The original HH equations for sodium channel (Table 3a, Fig. 5B), when used in simulations at 23–24 °C (the usual experimental room temperature) and corrected by a Q10 temperature coefficient equal to 3^12^, brought extremely fast and unrealistic activation and inactivation time constants for human sodium channels (Fig. 5C). By reducing the amplitude of the rate constants of *m* and *h*, as well as by slightly modifying the voltages and slopes parameters of the inactivation rate constants (Table 3b), it is possible to reach an overall initially acceptable fitting for the intensity-voltage curves of Na_V_1.5 (Fig. 5D).

**Table 2:** Experimental and simulated values for different protocols performed with HH and kinetic Markov-type channel models

Parameters	Experimental values*	Simulation values (kinetic)	Error value (kinetic)	Simulation values (HH)	Error value (HH)
Temperature	23–25 °C	24 °C		24 °C	
Sodium reversal potential	not available	65 mV		65 mV	
TC HMA (normalized conductance)	−34.5 ± 1.5 mV	−34.1 mV	1.61 × 10^−4^	−34.4 mV	1.39 × 10^−4^
TC Slope (normalized conductance)	−7.2 ± 0.6	−6.9		−7.2	
TC HMI for fast inactivation (normalized current)	−89.1 ± 1.6 mV	−89.5 mV	1.47 × 10^−5^	a) −88.4 mV b) −88.8 mV c) −89.3 mV	c) 1.14 × 10^−4^
TC Slope for fast inactivation (normalized current)	5.5 ± 0.4	5.4		a) 11.1 b) 5.5 c) 9.1	
Time constant of the recovery from fast inactivation (at −120 mV recovery voltage)	5.1 ± 0.9 ms	5.2 ms	2.30 × 10^−5^	a) 5.1 ms b) 0.2 ms c) 2.6 ms	c) 3.06 × 10^−6^
Time constant of the recovery from fast inactivation (at −110 mV recovery voltage)	12.5 ± 2.1 ms	11.0 ms (97%)	1.85 × 10^−7^	a) 6.7 ms (89%)	a) 1.62 × 10^−5^
Time constant of the recovery from fast inactivation (at −100 mV recovery voltage)	26.1 ± 3.8 ms	25.7 ms (87%)	7.99 × 10^−6^	a) 8.1 ms (73%)	a) 1.76 × 10^−5^
Time constant of the recovery from fast inactivation (at −90 mV recovery voltage)	47.9 ± 3.4 ms	43.2 ms (51%)	1.33 × 10^−6^	a) 8.5 ms (51%)	a) 1.84 × 10^−5^
First time constant of the recovery from slow inactivation	5.1 ± 0.9 ms	5.2 ms	1.04 × 10^−7^	c) 2.6 ms	c) 5.33 × 10^−7^
Fractional recovery with first time constant	78%	81%		82%	
Second time constant of the recovery from slow inactivation	596.3 ms	597.3 ms		c) 592.8 ms	
Fractional recovery with second time constant	22%	19%		18%	
Time constant of the development of slow inactivation	1.79 ± 0.11 s	1.78 s	7.17 × 10^−14^	c) 1.76 s	c) 7.85 × 10^−14^

TC: transient current; HMA: half maximal activation; HMI: half maximal inactivation; a) simulation values after setting the HH parameters to mainly reproduce the recovery from fast inactivation, or b) the steady-state availability, or c) a trade-off between them.

Error value is the square norm between data and dependent variable treated as continuous curves.

*Experimental values from^22^.

In the empirical fitting process, the reduction of both the time constants of activation and inactivation was mainly achieved by decreasing the amplitudes of rate constants. Also, the approximately correct sequence of activation and inactivation across the voltage clamps was obtained by shifting the *V*_*1/2*_ parameter of the backward rate constant of inactivation and by modifying the slopes of both rate constants of inactivation (Table 3b).

**Figure 6:**
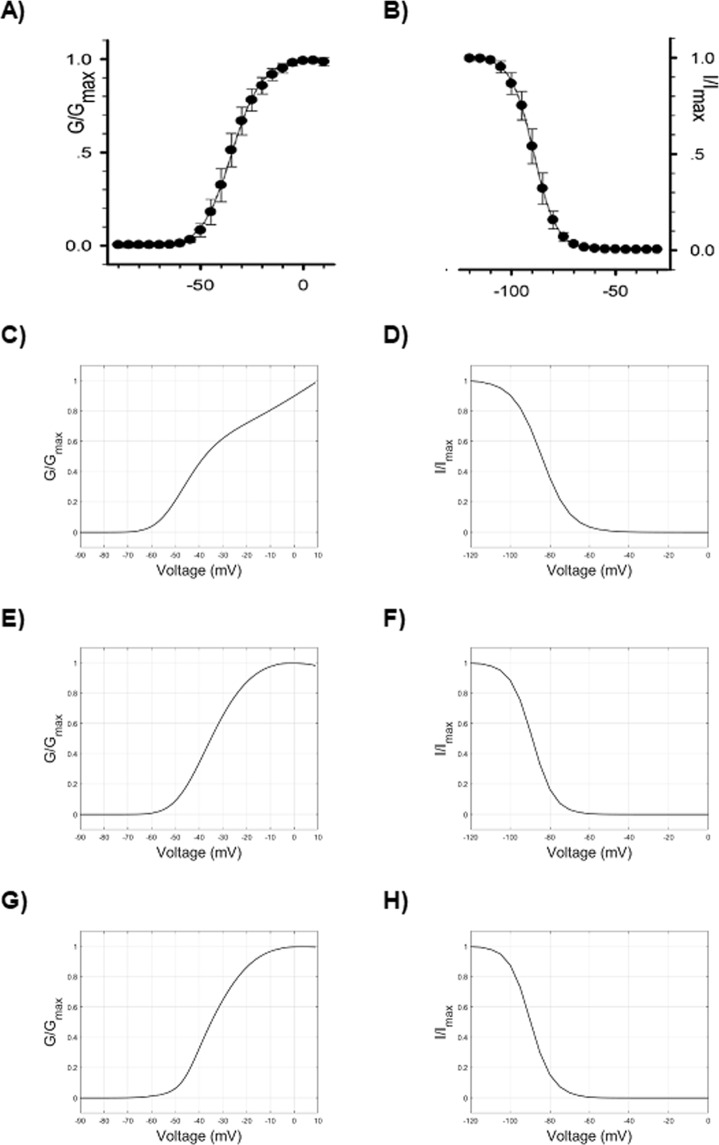
Voltage/Normalized conductance relationship during activation (left column) and Voltage/Normalized current relationship during fast inactivation (steady-state availability) (right column). (**A,B**) Experimental data^22^. (**C**) Simulated values of Voltage/Normalized conductance relationship during activation obtained by the HH model before parameters tuning (Table 3b). (**D**) Steady-state availability before parameters tuning (Table 3d) in the HH model. (**E**) Simulated values of Voltage/Normalized conductance relationship during activation obtained by the HH model after parameters tuning (Table 3c). (**F**) Steady-state availability after parameters tuning (Table 3e) in the HH model. (**G,H**) Simulated values obtained by the kinetic model.

The voltage dependence of normalized conductance in Na_V_1.5 has flat or slightly decreasing values along its course for the most depolarized values (starting from 0 mV, approximatively; Fig. 6A), while the corresponding values obtained with the just modified HH equations displayed an increasing course (Fig. 6C).

The activation-voltage relationship is mainly sustained by the reciprocal interaction of voltage dependence of α_*m*_ and *β*_*m*_, which gives rise to the voltage dependence of *m*_*∞*_, as depicted on Fig. 1C.

However, the voltage dependence of the activation peak can also be modified by acting on the rate constants of inactivation, and the correct (experimental) course of activation can be better approximated by modifying the slope and increasing the amplitude of *β*_*h*_, which yields a smaller value of *h* (that is, greater inactivation) for more depolarized voltages. In addition, after tuning the parameters of α_*m*_ and *β*_*m*_ (Table 3c), a modelled curve much more similar to the experimental one can be obtained (Fig. 6E; *V*_*1/2*_ = 34.7 mV, *k* = −7.2).

**Figure 7:**
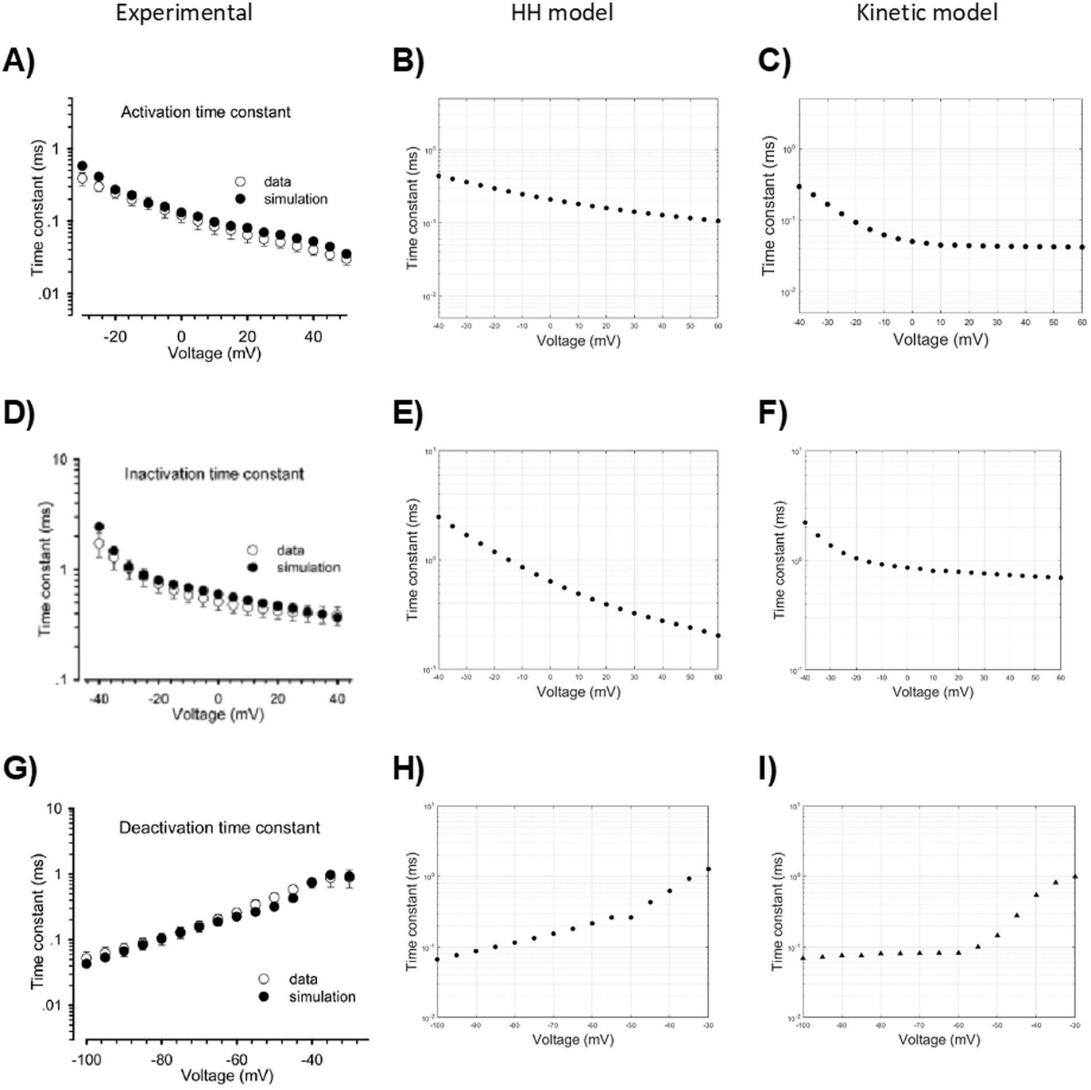
Time constants of activation (upper row), inactivation (middle row), and deactivation (bottom row) in function of voltage. Experimental values^22^ (left column), simulated data from a HH model (central column) and from a kinetic Markov-type model (right column).

The activation and inactivation time constants (Fig. 7A–F) were similarly fitted in the above passages by tuning the same parameters. Both were derived from the Eq. (3c): the activation time constant was computed at different voltages on segments between the onset and the activation peak, while the inactivation’s one was computed on segments between the activation peak and the return to the resting potential (decay from activation). It is worth mentioning here that the decay from activation is a first measure of inactivation.

#### Kinetic model

The transition rates of the kinetic Markov-type model were set in order to make all channels be in the C1 state at polarized resting potential (Fig. 3A,B; Table 4). By increasingly stepping the voltage towards more depolarized values, a progressively increasing fraction of channels moves to open conducting state (O1) through the second closed state (C2), due to increasing values of the transition rates between C1, C2 and O1. The forward and backward transition rates between C1 and C2, and between C2 and O1 determine the fraction of channels in O1 state and the time needed to reach this condition. However, since the O1 to I1 transition rate is always greater than zero and the I1 to O1 transition rate is remarkably smaller than the O1 to I1 transition rate, the fraction of channels in O1 state moves to I1 (that is, inactivates), with a velocity set by the O1 to I1 transition rate. The O1 to I1 transition, indeed, can be considered irreversible and, therefore, the channel does not rest in O1, moving instead to the inactivated state(s). In this kinetic model, the inactivated state follows the open state and is not independent from it, which is a behaviour in agreement with real data^13^.

**Table 3:** Parameters of the HH model.

		*m*	*h*	
**a**				
α	A	0.1	0.07	
	V_1/2_	−40	−65	
	k	10	−20	
β	A	4	1	
	V_1/2_	−65	−35	
	k	−18	−10	
**b**				
α	A	**0.03**	**0.002**	
	V_1/2_	−40	−65	
	k	10	**−10**	
β	A	**0.3**	**0.5**	
	V_1/2_	−65	**−25**	
	k	−18	**−15**	
**c**				
α	A	**0.02**	0.002	
	V_1/2_	**−48**	−65	
	k	**6**	−10	
β	A	**0.2**	**1.2**	
	V_1/2_	**−60**	**20**	
	k	**−100**	**−23**	
**d**				
α	A	**0.015**	0.002	
	V_1/2_	−**54**	−65	
	k	6	−10	
β	A	**0.4**	**0.9**	
	V_1/2_	**−70**	20	
	k	**−35**	**−25**	
**e**				
α	A	0.015	**0.0005**	
	V_1/2_	−54	**−67**	
	k	6	**−7**	
β	A	0.4	0.9	
	V_1/2_	−70	20	
	k	−35	−25	
**f**				
α	A	0.015	**0.0035 [0.004]**	
	V_1/2_	−54	**−65 [−75]**	
	k	6	**−25 [−16]**	
β	A	0.4	0.9	
	V_1/2_	−70	20	
	k	−35	−25	
***g***				
		***m***	***h***	***s***
α	A	0.015	0.004	**0.000092**
	V_1/2_	−54	−75	−**65**
	k	6	−16	−**55**
β	A	0.4	0.9	**0.00011**
	V_1/2_	−70	20	−**20**
	k	−35	−25	−**10**
**h**				
		***m***	***h***	***s***
α	A	0.015	0.004	**0.0001**
	V_1/2_	−54	−75	−65
	k	6	−16	−**50**
β	A	0.4	0.9	0.00011
	V_1/2_	−70	20	−20
	k	−35	−25	−10

In bold the parameters progressively tuned, following the electrophysiological protocols simulations, in order to fit the experimental data.

In squared parentheses (3f): trade-off parameters, chosen to account for both the steady-state availability and the recovery from fast inactivation.

The proposed Markov-type kinetic model features a multi-step activation sequence, according to the experimental data^11^, which is based on two closed states before the open one, as they are, indeed, the minimum number of states allowing to replicate the activation curves and the voltage-dependence of normalized conductance (Fig. 5F, Fig. 6G, and Table 4). In fact, adopting a single closed state results in an excessively small, unrealistic progression of the activation time constant, as it can be observed in Fig. 5E. The open-to-inactivated transition (O1I1) contributes to the normalized conductance-voltage relationship and mainly sets the decay from activation.

### Deactivation

#### HH model

**Figure 8:**
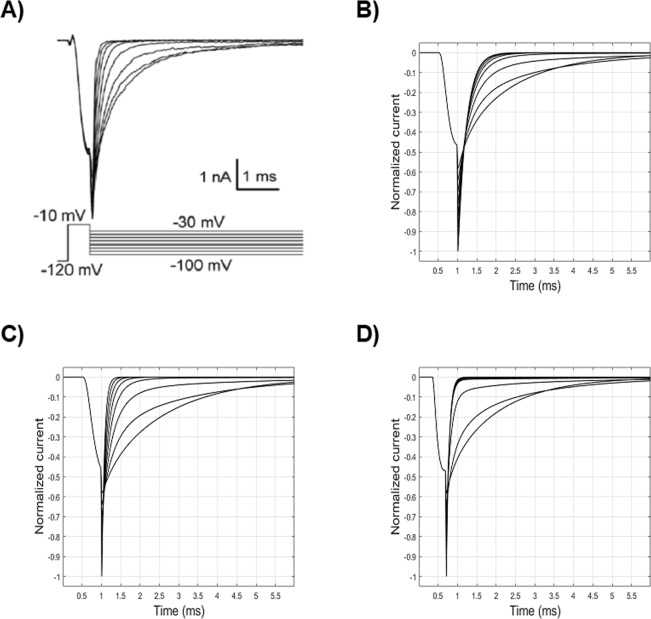
Current curves following the deactivation protocol. (**A**) Experimental values^22^. (**B**) Simulated values with the HH model before parameters tuning. (**C**) Simulated values with the HH model after parameters tuning. (**D**) Simulated values by means of the kinetic Markov-type model.

Tail currents (Fig. 8A), evoked by a brisk repolarization after a very short depolarization pulse (before substantial channel inactivation), can be fitted to a monoexponential function (Eq. 3d), whose time constant reduces progressively for more hyperpolarized test stimuli (Fig. 7G). They are mainly modelled by the return of *m* at low values during repolarization. Following the previous adjustment of HH model parameters (Table 3c), the simulated curves exhibit too long time constants for more hyperpolarized test stimuli (Fig. 8B). By varying the parameters of *β*_*m*_ (Table 3d), which is responsible for low values of *m*_*∞*_ at hyperpolarized values, the model is able to reach fairly similar time constants in the hyperpolarized range (Fig. 8C). However, due to changes in the activation-voltage relationship induced by the *β*_*m*_ variations, slight adaptations of α_*m*_ and *β*_*h*_ parameters (Table 3d) are needed to achieve again an acceptable fitting of the activation relationship (*V*_*1/2*_ = 34.4 mV, *k* = −7.2).

#### Kinetic model

The transition from the open (O1) to the closed (C2) state (Fig. 3B, *dashed red line*) is mainly responsible for the tail currents in the kinetic Markov-type model. In particular, a fair approximation of experimental values (Figs. 8D and 7I) can be reached by finely tuning the voltage dependence of the O1C2 transition rate in the more hyperpolarized voltage range. In that range, the prevalent fraction of closed channels is assured by the lower values of the corresponding C2O1 transition (Fig. 3B, *solid blue line*).

### Fast inactivation

#### HH model

The voltage dependence of normalized current during fast inactivation (steady-state availability) represents the fraction of ion channels not inactivated (i.e., available to activation), at different steady-state voltages. It is mainly given by the interplay between α_*h*_ and *β*_*h*_, which carries out *h*_*∞*_ values close to 1 at hyperpolarized voltages and close to 0 at depolarized voltages, as depicted in Fig. 1B,D. Experimental values of steady-state availability in Na_V_1.5 are depicted in Fig. 6B. The HH parameters values set until now (Table 3d) reproduce an availability curve with fairly correct morphology (Fig. 6D), although shifted towards overly depolarized values (*V*_*1/2*_ = −84.1 mV), and excessively slanted (*k* = 7.1). In order to shift the curve towards more hyperpolarized value (i.e. inactivation develops earlier, at more hyperpolarized voltages), it can be useful to reduce the amplitude of α_*h*_, while maintaining the value of *β*_*h*_ unchanged, in order to not modify the reached good approximation of the activation curve. In addition, we can also try to modify the slope of α_*h*_ to obtain a quicker transition to inactivation.

By adjusting the parameters of α_*h*_ (Table 3e) it is possible to obtain a reasonable approximation (*V*_*1/2*_ = −88.8 mV, *k* = 5.5) to the experimental curve (Fig. 6F). The parameters of the steady-state availability curve (that is, hemi-inactivation and slope) represent the second measure of the inactivation, distinct from the decay from activation.

It is worth noting that α_*h*_ mainly governs the steady-state availability (more hyperpolarized values), and *β*_*h*_ is responsible for the activation relationship (for more depolarized voltages, as seen above), which also affects the decay from activation.

In addition, as understood by the steady-state availability curve, the value of *h* at hyperpolarized voltages (more negative than −100 mV) should be 1. On the other hand, a quick inactivation develops at voltages around −90 mV, well before the start of activation (Fig. 6A). As shown, both the phenomena can be addressed by finely tuning the interplay between α_*h*_ and *β*_*h*_.

#### Kinetic model

In the electrophysiological protocol for studying the steady-state availability (Fig. 4C), long enough conditioning stimuli of increasing voltage are able to set an increasing fraction of channels into an inactivated state, and the following test stimulus samples the fraction of channels available for activation (not inactivated). What is interesting and crucial here is that even conditioning stimuli below the activation threshold are able to promote the transition to an inactivated state. Therefore, at subthreshold depolarizations a fraction of channels transits from the closed state to the inactivated state, without passing through the open state. While the HH formalism does not explicitly model open, closed and inactivated states, the proposed kinetic Markov-type model can specifically address this mechanism by tuning the C1 to I1 transition (C1I1, Fig. 3D, *dashed red line*). The resulting curve for the kinetic model is displayed in Fig. 6H, and the corresponding values of the C1I1 parameters are displayed on Table 4.

### Recovery from fast inactivation

#### HH model

The recovery from inactivation (or repriming) is sampled by a highly depolarizing conditioning pulse followed by a variable time interval of repolarization (from tenths to hundreds of milliseconds, in the case of Na_V_1.5), followed by a second depolarizing pulse, which tests the fraction of channels recovered from the inactivation (following increasing time intervals of repolarization) (Fig. 4D). During the first pulse of depolarization, *m* quickly increases (up to 0.8, in our simulation) (Fig. 1C), while the *h* value, which starts at a value of 1 (polarized resting voltage, that is no inactivation), decreases to a value of 0 (Fig. 1D) with a slower time constant compared to *m*. This provides the mechanism for the peak of sodium current. At the steady-state of activation-depolarization, which in Na_V_1.5 is reached within about 10 ms after the start of the conditioning pulse, *h* is equal to 0 (complete inactivation) and *m* is equal to 0.8 in our simulation, which produces no sodium current at all. The following repolarization, due to the higher value of *β*_*m*_, quickly sets the *m* value to 0 and, with a slower time course, the *h* value to 1 (absent inactivation).

By repolarizing the membrane with increasing intervals, the recovery from inactivation kinetics are sampled, which are mainly carried out by the voltage dependence of α_*h*_, provided that *β*_*h*_ has values close to 0 at −120 mV (the repolarization voltage), and that the kinetic of *m* is much faster (Fig. 1E).

**Figure 9:**
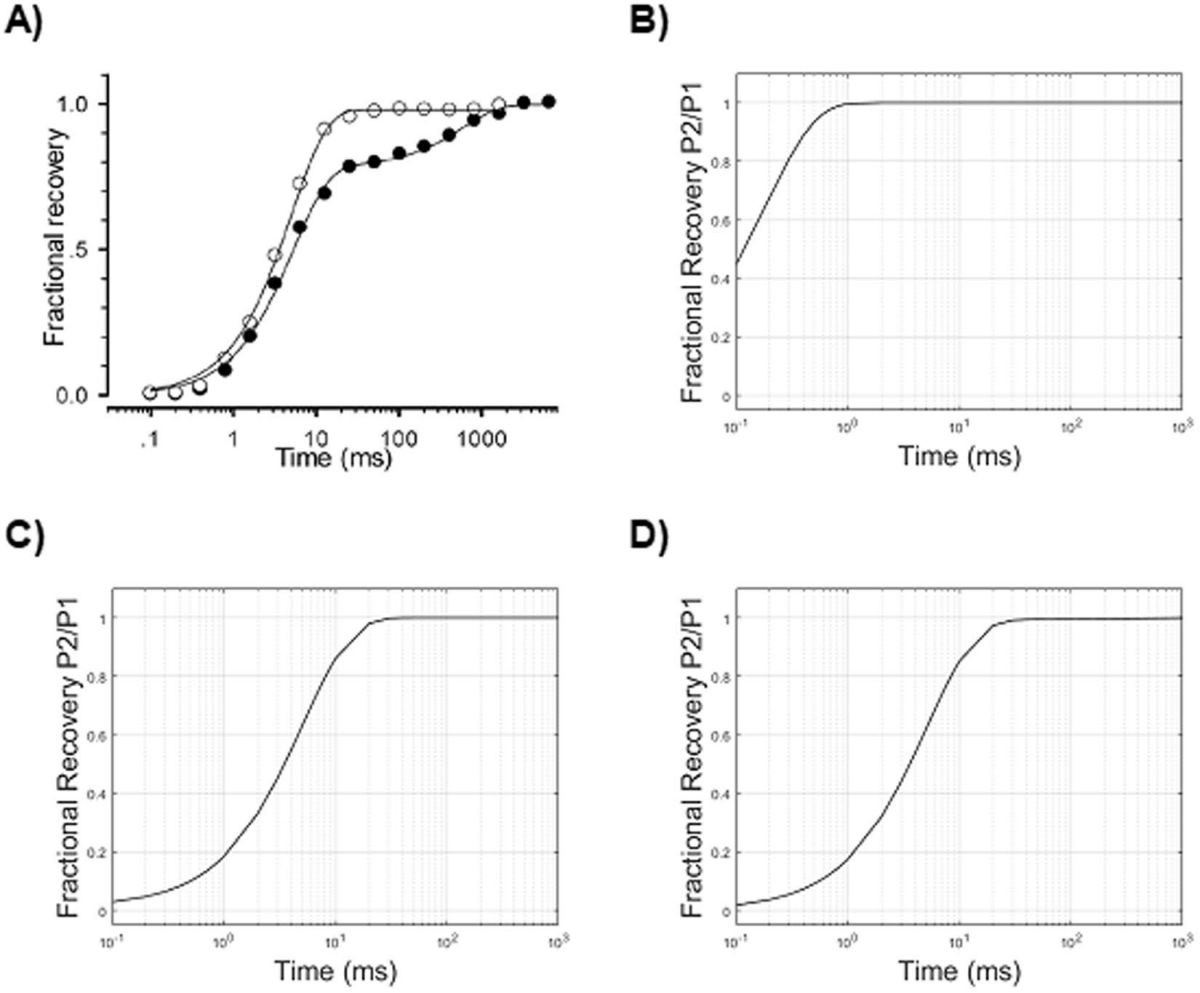
Recovery from fast inactivation: the fraction recovery (proportion of not inactivated channels after a delay from the first conditioning test) is reported as the ratio P2/P1 (P1: peak current intensity after the conditioning depolarization; P2: peak current intensity after the test depolarization) in function of time intervals of repolarization (logarithmic axis). (**A**) Experimental values (*empty circles*)^22^. (**B**) Simulated values with the HH model before parameters tuning (Table 3e). (**C**) Simulated values with the HH model after parameters tuning (Table 3f). (**D**) Simulated values obtained by the kinetic Markov-type model.

**Figure 10:**
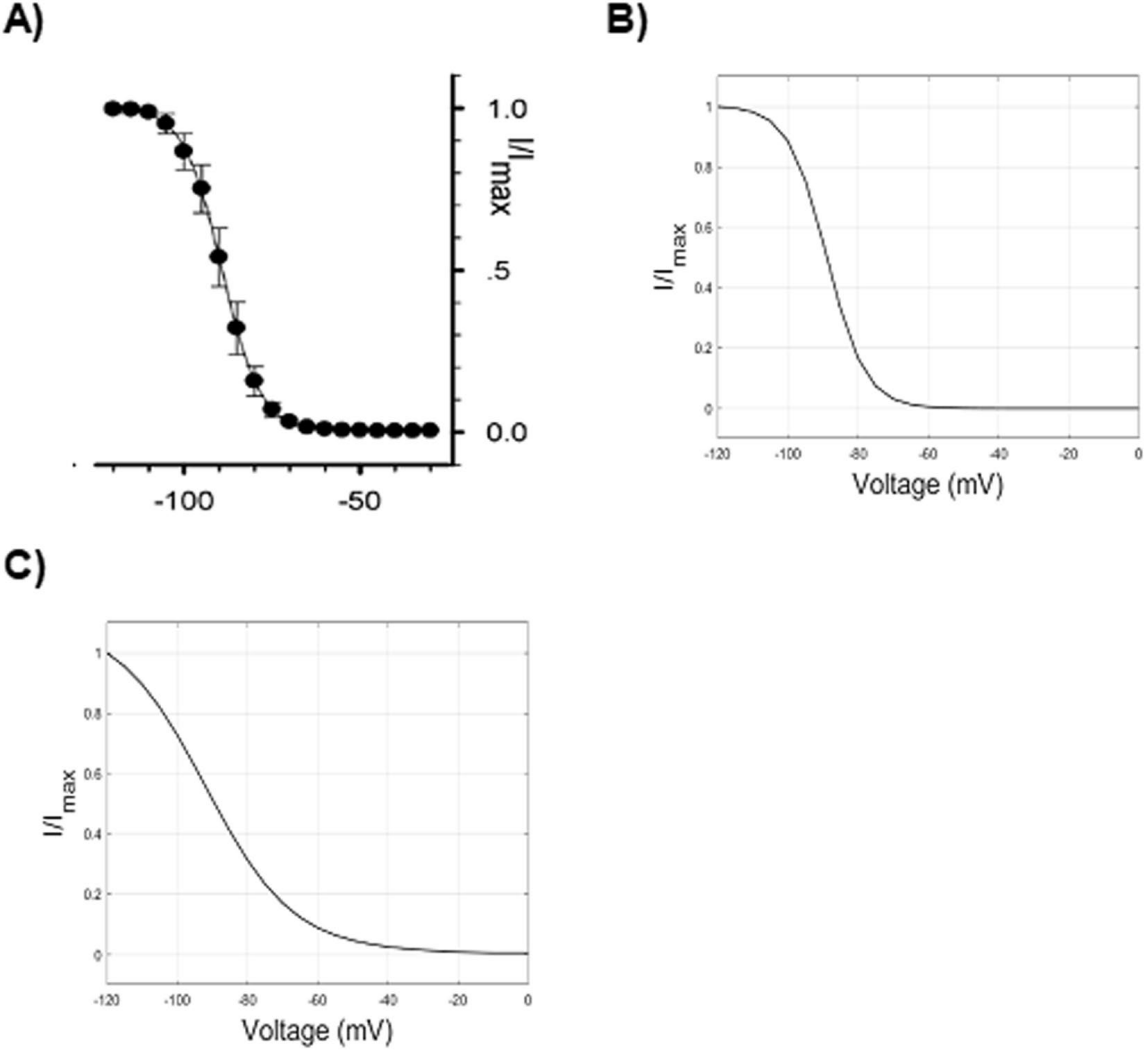
Effect of optimization of recovery from fast inactivation on steady-state availability in the HH model. (**A**) Experimental values of Voltage/Normalized current relationship following steady-state availability protocol^22^. (**B**) Simulated values of Voltage/Normalized current relationship following steady-state availability with the HH model before tuning parameters (namely α_*h*_) in order to optimize the recovery from inactivation (Table 3e). (**C**) The same relationship after parameters tuning (Table 3f).

The recovery curve (Fig. 9B), obtained by using the HH parameters set as above (Table 3e), shows an unrealistic substantial recovery after 0.1 ms of repolarization, and a much shorter recovery time, as compared to the experimental recovery curve. By reducing the amplitude of α_*h*_ (Table 3f), a more correct fitting can be obtained (Fig. 9C). However, since the forward rate constant of inactivation α_*h*_ also directly affects the fast inactivation curve, as shown above, the modified value does not provide a good fitting of the steady-state availability curve anymore (Fig. 10C, to be compared to Fig. 10A,B). Thus, the voltage dependence of α_*h*_ in HH models affects both the recovery from inactivation and the steady-state availability. This results in a conflicting limitation for the parameter optimization of the model. In other words, the detailed reproduction of the steady-state availability affects the accuracy of the recovery from inactivation. In Table 2, an example of this conflicting behaviour of the model is provided: the parameters of α_*h*_ have been set to preferably reproduce in detail the repriming (a), or the steady-state availability (b); alternatively, a trade-off can be made, by choosing parameters which carry out the closest, yet not detailed, approximation for both the electrophysiological features (c).

In addition, even when the HH model is set to reproduce the time constant of repriming, it fails when different voltages of repolarization are chosen (Table 2). In order to fix this flawed behaviour, one should also modify *β*_*h*_, which has relevant effects (i.e., it affects the time constant of recovery) in the hyperpolarized range (around −100 mV) when the value of α_*h*_ is small. However, each variation of *β*_*h*_, as mentioned above, also changes the voltage dependence of activation, which, in this case, results not amendable by further modifying α_*m*_ and *β*_*m*_ (data not shown).

Finally, it is worth mentioning that the recovery from inactivation provides a third measure of the fast inactivation.

#### Kinetic model

Recovery from inactivation can be easily set in detail by tuning the transition from the inactivated state I1 to the closed state C1 (I1C1, Fig. 3D, *solid blue line*), a way which is currently considered much more alike to the real biophysics of the channel (Fig. 9D; Table 4). In addition, no conflict arises with the electrophysiological behavior evoked by the steady-state availability protocol, which is mainly governed by the C1I1 transition, as shown above.

### Development of slow inactivation

#### HH model

In addition to fast inactivation, sodium channels also exhibit a slower inactivation, which was unrecognized when the HH model was developed. The development of the slow inactivation can be sampled by the protocol depicted on Fig. 4E.

To reproduce the slow inactivation kinetics by means of the HH formalism, an additional third particle (or gate) has to be considered in the conductance equation (Eq. 1c), which is usually named *s* and carries rate constants formally similar to those of *h*,

4 $$g={g}_{max}\times {m}^{3}\cdot h\cdot s$$

**Figure 11:**
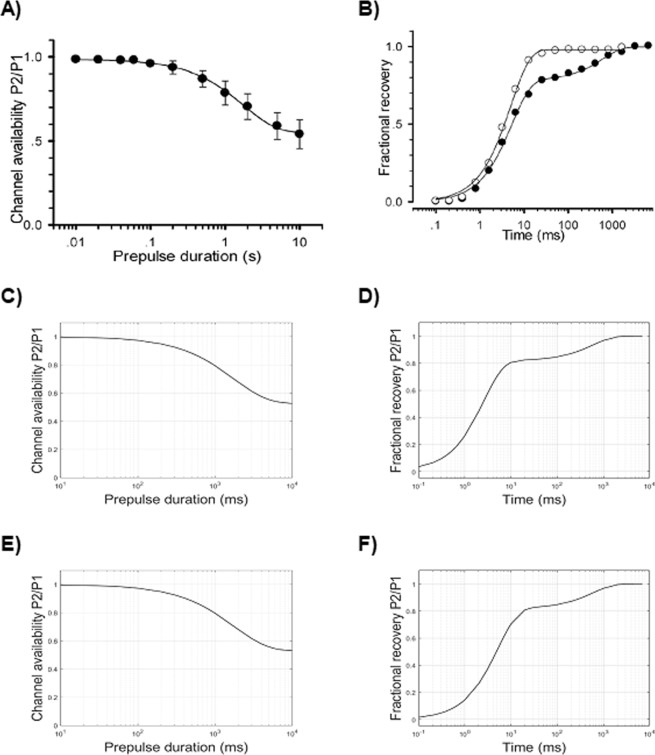
Development of slow inactivation (left column) and recovery from slow inactivation (right column). Abscissae in logarithmic scale of time intervals. (**A**) Development of slow inactivation, and (**B**) recovery from slow inactivation (solid circle) in the experimental setting^22^. (**C**) Development of slow inactivation (Table 3g), and (**D**) recovery from slow inactivation (Table 3h) in the HH model. (**E**) Development of slow inactivation, and (**F**) recovery from slow inactivation in the kinetic Markov-type model.

In this case, the development of slow inactivation is mainly modelled by tuning the voltage-dependence of the α_*s*_ rate constant (which, similarly to *h*, controls the transition from 1 to 0 of *s*). Also, it should be considered that, for the depolarization voltage (P2) used in the protocol (that is, −20 mV), even *β*_*s*_ must be tuned (that is, not 0 value), in order to obtain the correct proportion of non-slowly-inactivating channels even at longer durations of P1. Therefore, after adding the *s* factor to the HH model and tuning its parameters (Table 3g), a fairly good approximation of the development of slow inactivation can be reached (Fig. 11C). Note that, for this simulation, an intermediate setting of α_*h*_ has been adopted to provide a trade-off between the best fittings of steady-state availability and recovery from fast inactivation.

#### Kinetic model

The development of slow inactivation in the kinetic Markov-type model is easily approximated by tuning the parameters of I1 to I2 transition (Table 4, Fig. 3E, *solid blue line*). Analogously to the HH model, to reach the correct proportion of not-slowly-inactivating fraction of the channels, even the I2 to I1 transition should be tuned, in a way that at −20 mV (the conditioning voltage) its value is different from 0. The obtained modelled development of slow inactivation is depicted in Fig. 11E.

### Recovery from slow inactivation

#### HH model

Similarly to the fast inactivation, also the recovery from slow inactivation is sampled by means of a double pulse protocol (Fig. 4E), in which the first depolarizing conditioning pulse (P1) is followed by a repolarizing time interval of increasing duration (Δt, from 0.1 s to 10 s), followed by a short probing depolarization (P2). The difference with the fast inactivation protocol is that P1 is much longer (1000 ms, compared to 100 ms). By adopting this protocol, a recovery time course develops with two time constants of recovery, the first and faster one for the kinetics of fast inactivation, and a slower one for the slow inactivation repriming. By tuning the slow inactivation (*s*) rate constants (Table 3h), a good agreement with the recovery time constant of slow inactivation can be obtained (Table 2 and Fig. 11D). Note that the time constant of recovery from fast inactivation is not correct because we adopted, for these simulations, an intermediate setting of α_*h*_ (Table 3h).

#### Kinetic model

The recovery from slow activation can be easily reproduced in detail by finely tuning the I2I1 transition (Fig. 3E, *dashed red line*). The low values of the amplitude parameter of I1I2 and I2I1 stand for the slow processes of entry into and recovery from this kind of inactivation. The resulting curve of repriming of the slow inactivation is depicted in Fig. 11F).

### Channel models implementation in a standard neuron model

This subsection is aimed at assessing the computational burden of the proposed channel model (compared to that of the HH model) and it is only intended as a non-exhaustive proof of its suitability to be implemented in computational models of single neural cells. To this aim, we exploited a reduced and simple neuron computational model, previously developed by Dodge and Cooley^32^. Due to the limited purpose of this simulation, no in-depth exploration of the implementation has been performed, and also a series of approximations, yet not realistic, have been adopted (e.g., the implementation of the heart sodium channel in a spinal motoneuron model, even though Na_V_1.5 has been also detected in the central nervous system^24^).

The model developed by Dodge and Cooley is a reduced model of spinal motoneuron equipped with sodium, potassium and leakage conductances and comprises a soma, an equivalent dendrite, an initial segment, a myelinated segment, a node, and an extra unmyelinated segment. In order to assess the suitability and versatility of our models, we simply substituted the original HH sodium conductance mechanism with either the modified HH model or the kinetic Markov-type one, tuned with the parameters specified on Tables 3h and 4, respectively.

Apart from the substitution of the original sodium conductance with our Na_V_1.5 models, we only set the resting potential (and in turn the leakage equilibrium potential) to −100 mV, instead of −80 mV, to take into account the different kinetics of Na_V_1.5 inactivation. All other biophysical and structural parameters of the neuron model were kept unaltered. When stimulated by a virtual electrode located into the soma, the neurons models equipped with the modified HH and Markov-type kinetic channel models proved capable to fire spikes, although the action potentials showed a wider duration and an increased threshold of the current stimulus (~150 nA versus ~65 nA), compared to the Dodge and Cooley model (see Supplemental Files, Fig. S1A–C).

In order to compare the computational load between the models, a 3000 ms long simulation was performed, in which a train of electrical stimuli (2800 ms long, with an amplitude of 160 nA and a frequency of 25 Hz) was delivered to the soma. In this simulation, a time step of integration of 0.001 ms was chosen, and ten runs were performed for each channel model.

Despite our expectations, the neuron model equipped with the HH channel model completed the simulations only about 2 s before the one equipped with the kinetic Markov-type channel model (34.19 ± 0.34 s versus 36.38 ± 1.43 s). Because of the lower computational load of the HH formalism we would have expected a greater difference. The original Dodge and Cooley model, equipped with channels built according to the HH formalism, completed the runs in 31.50 ± 0.14 s (Table 5).

**Table 4:** Parameters of the kinetic Markov-type model.

	*A*_*hyper*_	*V(1/2)*_*hyper*_	*k*_*hyper*_	*A*_*dep*_	*V(1/2)*_*dep*_	*k*_*dep*_
*C1C2*	—	—	—	8	−16	−9
*C2C1*	2	−82	5	8	−16	−9
*C2O1*	—	—	—	8	−26	−9
*O1C2*	3	−92	5	8	−26	−9
*O1I1*	8	−50	4	6	10	−100
*I1O1*	0.00001	−20	10	—	—	—
*I1C1*	0.35	−122	9	—	—	—
*C1I1*	—	—	—	0.04	−78	−10
*I1I2*	—	—	—	0.00018	−60	−5
*I2I1*	0.001825	−88	31	—	—	—

**Table 5:** Running time values.

	HH model, 2 gate variables (*m, h*)	HH model, 3 gate variables (*m, h, s*)	Kinetic model
Time (s ± SD)	31.50 ± 0.14	34.19 ± 0.34	36.38 ± 1.43
Ratio to the HH model, 2 gate variables	—	1.09	1.15
Ratio to the HH model, 3 gate variables	—	—	1.06

## Discussion

Our study recognizes some critical limitations of the HH formalism in modelling the known complexity of Na_V_1.5 macroscopic currents, and shows how a simplified kinetic Markov-type model is better suited to approximate in detail these currents. It also provides a practical guide to a procedural optimization of simplified kinetic models, which are shown to exhibit a computational load comparable to that of the HH model.

The need to develop models of ion channels able to reproduce in detail more recent electrophysiological data has been for long recognized^10,17^. On the other hand, both theoretical^11,13,20^ and practical^15,16^ limitations of HH models have been already reported. Among these, the HH model hypotheses of independent gating particles, the calculated time constant of deactivation, the behaviour of the gating current, have been proven to be inaccurate^33^.

In addition, here we demonstrate a critical practical limitation of the HH model which, to the best of our knowledge, has not been previously put explicitly forward. The steady-state inactivation and the recovery from inactivation have to be set in HH models by tuning an identical and unique parameter, the voltage dependence of α_*h*_. Thus, the detailed reproduction of the two electrophysiological behaviours results in a conflicting optimization limit.

In one of their seminal papers^34^, Hodgkin and Huxley devised three electrophysiological protocols to specifically study the behaviour of the (fast) inactivation of the voltage-gated sodium channel of *Loligo*. The protocols (Fig. 4C–E), with few modifications, would have been later known as steady-state availability, recovery from inactivation (or repriming) and development of inactivation, and, as for the entire Hodgkin and Huxley research, they set the framework for all following studies on the subject. In that paper, poor resolving power of the material (*Loligo* sodium channel has time constants of entry into inactivation - from closed states - close to the time constants of decay from activation) likely prevented the Authors to recognize that entry into inactivation can proceed along different mechanisms (namely from closed or open states). Consequently, they suggested only one time constant (named *τ*_*h*_) to describe the inactivation processes^12,34^. Thus, in HH models *β*_*h*_ (Fig. 1B) usually sets the decay from activation (due to its high value in depolarizing range) and α_*h*_, which properly establishes the recovery from inactivation, must be also used for providing the inactivation in a less depolarized range.

For the proposed Markov-type kinetic model, instead, each feature can be set independently by tuning different parameters: the decay from the activation via the O1I1 parameters, the recovery from activation via the I1C1 parameters, the steady-state availability via the C1I1 parameters.

### The phenomenological modelling

We recently described how a single simplified kinetic Markov-type model is able to reproduce in detail the macroscopic currents of all human voltage-gated sodium channels, Na_V_1.1 to Na_V_1.9^25^. Apart from the adherence to different experimental data, the proposed phenomenological model was also constrained by the parsimony of states and transitions, in order to build the simplest framework with minimal computational load and to provide a kinetic Markov-type model suitable for the implementation in large conductance-based neural networks.

When multiple and different neuronal models (even morphologically detailed), equipped with a variety of ion channels, have to be assembled in large and bio-realistic neural networks, the size and the computational load of the single channel models do matter. For example, a recent study^3^ modelled a neocortical volume of 0.224 mm^3^ containing about 45000 conductance-based neuronal cells, in which up to 10 types of HH ion channel models were variably inserted. The simulation was carried out on a massive parallel supercomputer of 120 CPU cores and required about 1 hour to simulate 1 second of real electrophysiological activity. In similar cases more complex and large channel models would exponentially increase the run-time, resulting hardly suitable to accomplish the simulation tasks. In these cases, simplified Markov-type kinetic models of ion channels would be of help in preventing unrealistic behaviours of neural models, by providing a more detailed description of the macroscopic currents with respect to HH models at a comparable computational cost.

The present study only deals with the macroscopic currents, and the proposed kinetic model is not intended to fit the data from biophysical studies of the channel, where single channel recordings are exploited to derive the subtle microscopic conformational changes of the pore-forming protein (e.g., ^21^), also taking into account the gating currents. In the present study the single states do not correspond to physical molecular states of the protein, and they should be rather considered as aggregates of molecular configurations operationally grouped into a set of distinct states separated by large energy barriers^8^. For example, our model condences the sequential closed states, before an open state can develop after a depolarizing step, in only two, at variance with other commonly adopted kinetic Markov models^20,22^, where a series of four (or more) closed states are hypothesized. The phenomenological behaviour of the channels, indeed, can be correctly reproduced by using only two closed states^25^.

The adopted step-by-step procedure and the manual optimization of the two channels models in close relation is here mainly adopted to show where and how the Markov-type model outperforms the HH model. On the other hand, alternative and automated optimizing methods exist, based on *ad hoc* algorithms^35–38^. They exploit global search methods as genetic algorithm, simulated annealing or others, to identify the optimal set of parameters of the mechanistic ordinary differential equations that simulate the dynamics of excitable cells. Mainly developed to search for optimal rate constants parameters of Markov models following single channel patch-clamp data, optimization algorithms have also been used to develop simplified Markov-type kinetic models of ion channels from macroscopic current recordings^38^.

### Theoretical and practical advantages of the Markov-type kinetic model

Unlike the HH formalism, Markov-type kinetic models try to embed the *a priori* knowledge derived from specific functional and structural studies on the biophysics of the channel, even in their simplified formulations. For example, they can account for the direct transition from closed to inactivated states^11^, for the inactivation passing through the closed states before re-activation^20^, for the multistep process of activation^11^, for the dependence of inactivation from activation^13^.

In addition, Markov-type kinetic models appear to be more adaptable and prone to incorporate novel or further channel features with respect to HH models. Indeed, as a general computational tool, they have been already exploited to simulate different neural phenomena, like ligand-gated channels or second messenger-activated channels, and they have been considered as a more general framework in the larger context of biochemical signal transduction^19^.

The results from the channels implementation in a neuron model show that the runtime of the kinetic model is only about 5% higher than that of the HH model, which seems to be an absolutely acceptable drawback, considering the advantages earned in terms of simulation accuracy and adherence to the biophysics of the ion channels.

### Predictions and suggestions from the kinetic model

Modelling studies can suggest slight variations of the canonical electrophysiological protocols to evoke uncommon dynamics of macroscopic currents with the aim of clarifying some lesser-known features of ion channels behaviour. For example, by adopting different levels of repolarization voltages during recovery from inactivation protocols^22^, a fractional recovery can be recognized (Table 2). Analogously, even the steady-state availability protocol could take advantage by adopting different levels of conditioning steady-state voltages.

As regarding the evaluation of time constants of activation and inactivation, we fitted the curve obtained by the activation protocol with the Eq. (3c), which involves a third power exponential to fit the activation segment of the curve, and a simple exponential for the inactivation (decay) segment. In the study carrying the experimental values we refer to^22^, a slightly different fitting procedure was adopted. Two separate fits for the activation and inactivation segments were performed, and both of them were fitted to a simple exponential, after (presumably) manually splitting the two segments of the curve.

Although this different fitting approach brought results that are slightly divergent (Fig. 7) from those by our experimental reference^22^, we chose it because: (a) it is the original procedure from the work by Hodgkin and Huxley^12^, (b) it provides the best fitting (minimum fitting error), (c) it is free from errors derived by manually splitting the curves.

In addition, in the present study a measure of the fitting error has been always reported. This is unfortunately an uncommon practice in ion-channels modelling, which, instead, could greatly improve the reliability of the comparison among models and their refinement.

### Limitations and future studies

The major limitation of the present modelling study comes from the lack of raw electrophysiological data. It would have been better to optimize the models based on raw clamping currents data instead of indirect relationships, such as normalized conductance-voltage relationship following activation voltage clamping, normalized current-voltage relationship following fast inactivation protocol, etc. Moreover, the possibility to compare the simulated data directly to the raw data would provide a more explicit and reliable model testing.

The availability of raw electrophysiological data in open access web repository with standard and shared format could overcome this limitation, common to other modelling studies, and would greatly improve the quality of ion channels modelling studies.

Modelling represents the challenging, continuous effort to approximate physical phenomena, by means of an established or hypothetical mathematical description of it. In this sense, it is a matter of progressive approximation and continuous updating, according to the deepening knowledge of the examined phenomena. The present study shows how the adoption of kinetic Markov-type models, along with the effort to limit their complexity (following the well-known ‘Occam’s razor’ principle), could also advantage the biologically inspired modelling of different kinds of ion channels, both voltage- and ligand-gated.

In following studies, further comparisons between HH and kinetic Markov-type models could be undertaken by exploiting more detailed cell models and neural networks. Previous studies^14^ already developed kinetic models of ion channels in order to fit experimental data not accounted for by HH models in detailed neuron cell models. Due to the limitations of HH formalism in modelling the inactivation kinetics, post-spike refractory times could show the most striking differences with kinetic Markov-type models.

## Supplementary information

Supplementary file

## Data Availability

The source code developed in NEURON 7.6 simulation environment, comprehensive of the NMODL description of channel models and all virtual experimental procedures, is available as a ModelDB^30^ entry (access number: 257747) (http://modeldb.yale.edu/257747).
