# Supplementary material for: Phenomenological models of NaV1.5. A side by side, procedural, hands-on comparison between Hodgkin-Huxley and kinetic formalisms
Source: Sci Rep. 2019 Nov 25;9:17493. doi: 10.1038/s41598-019-53662-9 (PMC6877610; doi:10.1038/s41598-019-53662-9)
Supplement: Supplementary file 1 — Supplementary file [file 41598_2019_53662_MOESM1_ESM.pdf]

Supplementary file

Phenomenological models of Na$_V$1.5.

A side by side, procedural, hands-on comparison between Hodgkin-Huxley and kinetic formalisms

Emilio Andreozzi, Ilaria Carannante, Giovanni D'Addio, Mario Cesarelli, Pietro Balbi

Figure S1

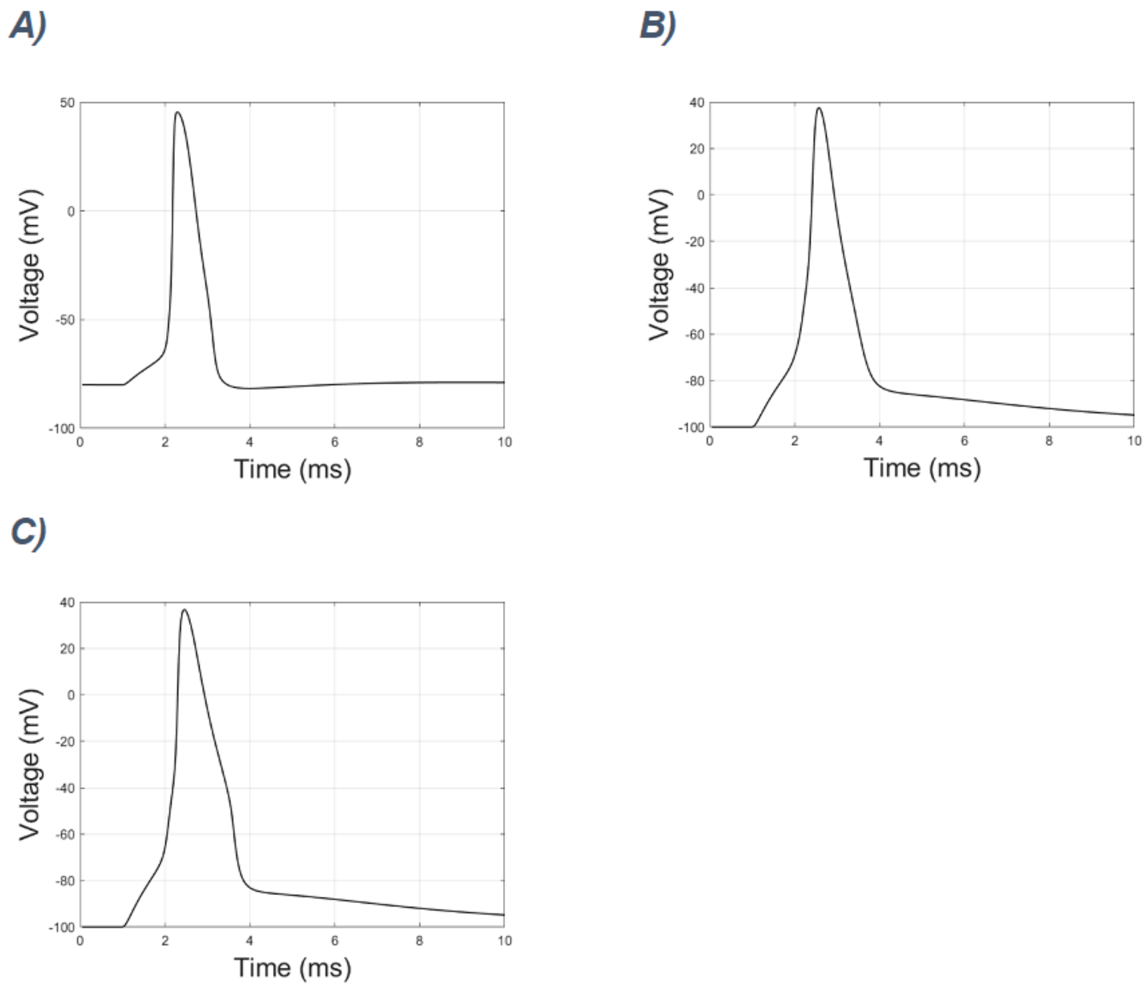

Figure S1. Action potential in a reduced spinal motoneuron[32] following a depolarizing somatic stimulus. A) Voltage displacement at the soma of the original cell model. B) Voltage displacement at the same location after substituting the original model of sodium channel with the Na$_V$1.5 model built according to the HH formalism, or C) according to the Na$_V$1.5 Markov-type kinetic model.

Figure S2

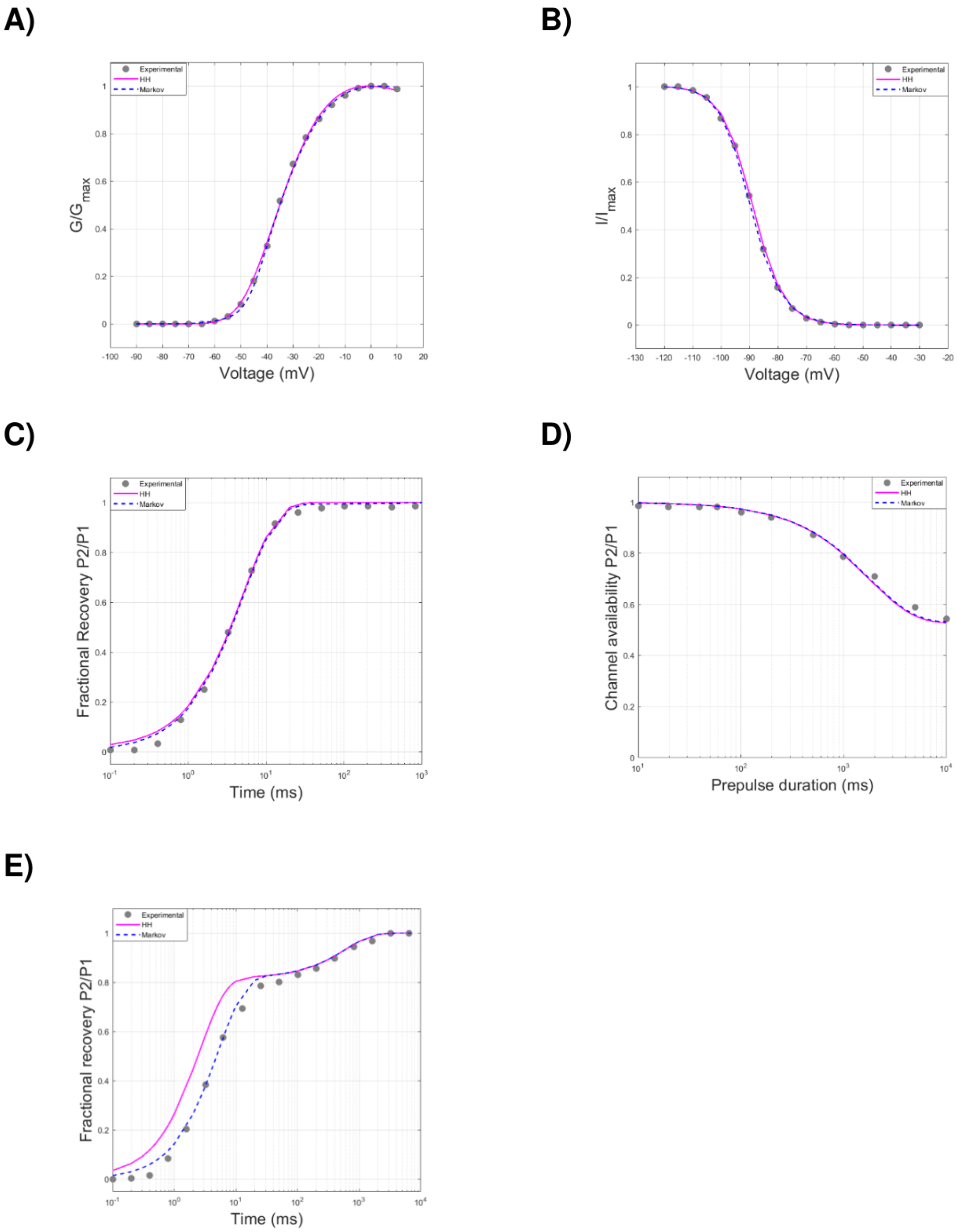

Figure S2

Overview of the experimental[22] data (gray circle) comparison with the simulated data, modelled according to the HH formalism (pink solid line) or the Markov-type kinetic formalism (blue dashed line). A) activation protocol; B) steady-state availability protocol; C) recovery from fast inactivation protocol; D) development of slow inactivation protocol; E) recovery from slow inactivation protocol.
